# Long Term Survival Analysis of Hepatectomy for Neuroendocrine Tumour Liver Metastases

**DOI:** 10.1155/2014/524045

**Published:** 2014-01-12

**Authors:** Tan To Cheung, Kenneth S. H. Chok, Albert C. Y. Chan, Simon Tsang, Jeff W. C. Dai, Brian H. H. Lang, Thomas Yau, See Ching Chan, Ronnie T. P. Poon, Sheung Tat Fan, Chung Mau Lo

**Affiliations:** Department of Surgery, The University of Hong Kong, Queen Mary Hospital, 102 Pokfulam Road, Hong Kong

## Abstract

*Background.* Liver is the commonest site for metastasis in patients with neuroendocrine tumour (NET). A vast majority of treatment strategies including liver directed nonsurgical therapy, liver directed surgical therapy, and nonliver directed therapy have been proposed. In this study we aim to investigate the outcome of liver resection in neuroendocrine tumour liver metastases (NELM). *Method.* 293 patients had hepatectomy for liver metastasis in our hospital between June 1996 and December 2010. Twelve patients were diagnosed to have NET in their final pathology and their data were reviewed. *Results.* The median ages of the patients were 48.5 years (range 20–71 years). Eight of the patients received major hepatectomy. Four patients received minor hepatectomy. The median operation time was 418 minutes (range 195–660 minutes). The median tumor size was 8.75 cm (range 0.9–21 cm). There was no hospital mortality. The overall one-year and three-year survivals were 91.7% and 55.6%. The one-year and three-year disease-free survivals were 33.3% and 16.7%. *Conclusion.* Hepatectomy is an effective and safe treatment for NELM. Reasonable outcome on long term overall survival and disease-free survival can be achieved in this group of patients with a low morbidity rate.

## 1. Introduction

Neuroendocrine tumours (NETs) comprise a wide range of neoplasm which originates from cells of nervous and endocrine systems. The most common sites of the disease include the small bowel, large bowel, and the pancreas. It can also arise from other parts of the body. Liver is the most common site of metastases for gastrointestinal NETs.

Upon the first diagnosis, 56%–93% patients were found to have synchronous neuroendocrine liver metastases (NELM) together with a primary tumour [1]. It was not uncommon that the metastases involve both lobes of the liver with diffuse manifestations. Only around 10% of the patients was eligible for liver resection upon discovery [2]. Although liver resection was considered to be the most effective treatment for NELM in terms of survival, not every patient was considered for hepatectomy due to the potential complications and even mortality. Unlike colorectal liver metastasis, hepatectomy for NELM had not been supported by large scale studies. Many published data involve small numbers of patients because resectable NELMs are not common.

In this study, we aim to analyze outcome of liver resection for NELMs with curative intentions.

## 2. Materials and Methods

From June 1996 to December 2010, 293 patients received hepatectomy for liver metastases at Department of Surgery, Queen Mary Hospital, The University of Hong Kong, Hong Kong. Twelve patients with NET liver metastases were included in the present study. All patients had liver resection performed with the histological diagnosis of neuroendocrine tumour liver metastases. Patients were followed up by a multidisciplinary team that consisted of surgeons and oncologists.

This was a retrospective designed study, but all the clinical data had been collected prospectively in a computerized data base recording the preoperative, perioperativel and postoperative information by a single research assistant. The use of data and the study was approved by the hospital institutional review committee.

Liver metastases were diagnosed by contrasted CT scan or contrasted MRI. Octreotide scan or positron scan (PET) with DOPA tracer would be performed before consideration of liver resection [3–6]. Upon diagnosis of liver metastases patients were assessed by consultant hepatobiliary surgeons for feasibility of liver resection. In this study, only patients with lesions that could be completely resected with curative intention were included. We did not perform debulking liver resection in this series.

Patients with (1) absence of extrahepatic disease as evidence by octreotide scan or PET scan, (2) lesions which can be resected completely and (3) good functional reserve with future liver remnant more than 30% estimated standard liver mass were selected for liver resection.

We did not perform radiofrequency ablation for NELM in this series.

Patients with unresectable diseases would be referred to the oncologist for consideration of non surgical liver directed surgery or systemic therapy.

All hepatectomies were performed by experienced hepatobiliary surgeons. Open approach was adopted in all twelve patients. Bilateral subcostal incision with midline extension was usually used for major hepatectomy. Intraoperative ultrasound was performed during laparotomy. Liver resection would be carried out only if curative hepatectomy was feasible. Cholecystectomy was then performed and the cystic duct was cannulated with an Fr 3.5 Argyle tube before major hepatectomy. Liver parenchymal transection was performed mainly with the cavitron ultrasonic surgical aspirator (CUSA). Haemostasis was achieved by electrocautery, argon beam, and suture. Pringle maneuver for hepatic inflow control was not routinely performed. Methylene blue leakage test was performed at the end of major hepatectomy to exclude biliary leakage after parenchymal transection. Abdominal drain was not routinely deployed.

Major hepatectomy was defined as removal of more than 3 anatomical sections.

The resected specimens were sent for histopathological examinations. The tumours were classified into low grade (<2 mitotic figures/50 hpf), intermediate grade (2–50 mitotic figures/50 hpf) and high grade (>50 mitotic figures/50 hpf) [7]. Immunohistochemical staining was performed in some patients who were operated on in a more recent period. High Ki-67 staining was defined as abnormal expression >5% [8].

Contrast CT scan was performed one month after the hepatectomy. The patients were followed up in our clinic at every 3 months with contrast CT reassessment at the first 2 years and every 6 months with contrast CT scan from third year after operations. Serum Chromogranin A (CgA) level and urine 5-hydroxyindoleacetic acid (5-HIAA) level would be checked in the clinic. Patients were followed up by the surgeons and the oncologist. Recurrence was defined as typical features presented on contrast CT/MRI scan on follow-up. Biopsy of the lesions would be performed if necessary. The overall survivals and disease-free survivals after the liver resections were analyzed.

## 3. Statistical Analysis

The baseline characteristics of patients were expressed as medians with range. The Mann-Whitney *U* test was used to compare continuous variables, and a chi-square test was used to compare discrete variables. Survival curves were computed using the Kaplan-Meier method and compared between groups by the log-rank test. Significance was defined as *P* < 0.05. All statistical calculations were made with the SPSS/PC+ computer software (SPSS, Chicago, IL, USA).

## 4. Results

The median age of the patient was 48.5 years (ranged 20–71 years). There were seven male patients and five female patients. Two patients (16.7%) were hepatitis B virus carrier. Five patients (41.7%) had co-morbid illness before hepatectomy. None of the patient presented with a functional NELM. The patients' demographic data is listed in Table 1. Four patients (33.3%) had minor liver resections for the NELM. Eight patients (66.7%) had major liver resections for NELM. The median blood loss was 1.29 L (ranged 0.2–7 L). The median operation time was 418 minutes (ranged 195–660 minutes). Pringle maneuver was performed in two patients. The median inflow control time was 40 minutes (ranged 20–40 minutes). The median hospital stay was 11 days (ranged 4–56 days). There was no hospital mortality. Three patients (25%) had complications after hepatectomy. Two patients developed pleural effusion after the surgery and one of them required pleurocentesis for symptomatic relieve. One patient had developed subphrenic collection and required percutaneous drainage. The operation details are listed in Table 2.

Six patients (50%) in our series had NELM from pancreatic origin; four patients (33.3%) had NELM from unknown origin; one patient (8.3%) had NELM from adrenal in origin and one patient (8.3%) had NELM from colon in origin. The median tumour size was 8.75 cm (ranged 0.9 cm–21 cm). Six patients (50%) had one tumour, one patient (8.3%) had three tumours, one patient (8.3%) had four tumours and 4 patients (33.3%) had multiple tumours. Eleven patients (91.7%) had R0 resection and one patient (8.3%) had R1 resection. The median resection margin from the tumour was 5 mm (ranged from 1 mm to 15 mm). Microvascular invasion was found in six patients (50%). One patient had high mitotic figure in the tumour. Five patients had low mitotic figures in the tumours. Three patients had >5% expression of Ki-67 in the tumours. The individual patient's performance is listed in Table 3.

The Median follow-up time was 52.8 months. The median survival was 52.8 months (range 4–57.8 months). The one year survival was 91.7% and the three year survival was 55.6%.

**Figure 1:**
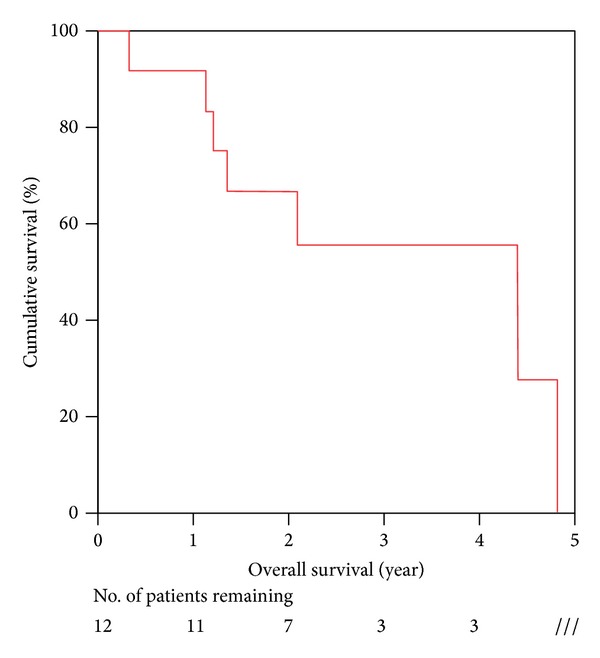
Overall survival of NELM patient after hepatectomy.

The survival curve is listed in Figure 1.

The median disease-free survival was 5.8 months (ranged 1 month–51 months). The one year and three year disease-free survival was 33.3% and 16.7%.

**Figure 2:**
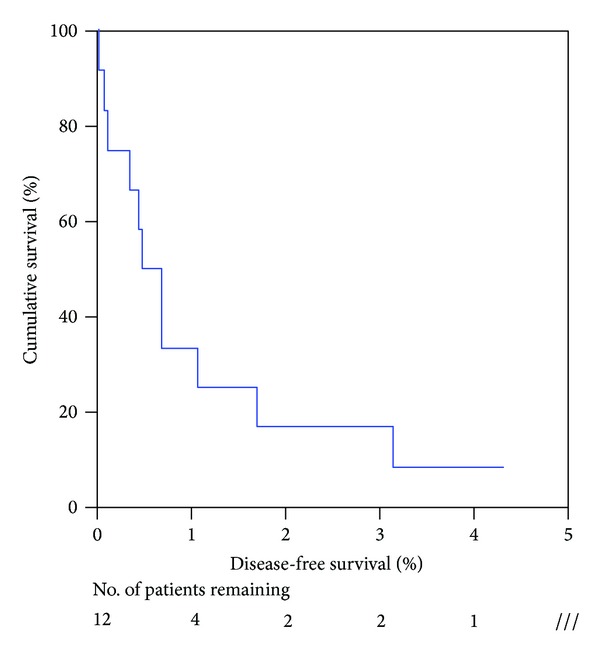
Disease-free survival of NELM patient after hepatectomy.

The disease-free survival is listed in Figure 2.

Ten possible factors that might affect the survival were included for univariate analysis and were listed in Table 4. None of these factors seemed to have adverse effect on overall survival.

## 5. Discussion

NELM was a rare disease when compared to colorectal liver metastases. The nature of history of NELM and the analysis of the best treatment modalities were based on small scale studies [9–13]. Compared to other metastatic adenocarcinoma of the liver, NELMs were thought to be more indolent due to the nature of the disease. But in reality, the 5-year survival of patients with NELMs on best supportive treatment ranged from 0% to 20% [1, 2, 14]. The prognosis remained dismal if no treatment was offered. A wide variety of treatment options were available including surgical resection, liver directed therapies, and systemic therapies.

Amongst these treatment options, liver resection seemingly was the most invasive approach. Nevertheless, with careful patient assessment, the mortality could be less than 5% even in patients with adverse underlying liver parenchymal disease like cirrhosis [15]. The safety of hepatectomy for noncirrhotic liver was even higher when carried out in high volume centers [2, 16–18].

In our institution, a thorough liver function evaluation was strictly followed before consideration of liver resection [19]. Patients with Child Pugh A liver function, adequate future liver remnant volume determined by preoperative CT volumetry and satisfactory indocyanine green (ICG) retention rate would largely guarantee the safety of liver resection. Improvements in technologies had also enhanced the safety of liver resection. The use of pulse spectrophotometry device allowed a rapid measurement of ICG retention within 6 minutes which is closely correlated to conventional ICG measurements [20]. Pulse spectrophotometry could also be performed during the peri-operative period. In addition, the use of CUSA and new energy devices could enhance the efficiency of liver transection by reducing the blood loss. Blood loss was identified to be an important factor predisposing to hospital mortality in liver resection. Careful administration of intravenous fluid during the operation could prevent venous congestion of the liver which could make liver parenchymal transection less difficult. When excessive bleeding was encountered, the selective use of Pringle Maneuver could be endorsed in an intermittent manner [21]. In many patients with NELMs, there were multifocal involvements in both lobes of livers. Mayo el at the reported that more than 47% of patients had more than 50% hepatic involvement by the metastases upon presentation [2]. The prognosis of a patient with diffuse liver metastases in general was poorer when compared to those with less extensive involvement. In view of this information, patients who had extensive liver metastases might not be referred for liver resection. In our study a more aggressive approach to NELM were adopted. Two-third of the patients in this series actually had diffuse liver metastases which end up in major hepatectomy. The best alternative treatment for diffuse NELM was intraarterial therapy (IAT). The most widely used modality was by transarterial chemoembolisation (TACE). Since most of the NELMs were rich in arterial supply, TACE could effectively deliver the chemotherapeutic agent to the targeted lesions. The femoral artery was catheterised under local anaesthesia. The right or left hepatic artery was usually super-selectively catheterized. An emulsion of cisplatin or doxorubicin with Lipiodol was introduced. After maximal drug was administrated to the tumour, embolization of the feeding artery by small gelatine-sponge pellets of 1 mm diameter would be introduced [22]. There were very little data on the outcome of TACE on non-functioning NELM. Mayo et al. showed that in 66 patients who had non functioning NELM with liver involvement >25%, none of the patients had complete tumour response by RECIST criteria. Only 6.3% of patients had partial tumour response by RECIST criteria. The median survival for this group of patient was 18.5 months [2]. In our study, despite the fact that more than 60% of our patient had multifocal NELM, performing major hepatectomy in this group of patient by removing more than 50% of the liver volumes leaded to a median survival of 52.8 months.

The short term surgical outcome for major liver resection and minor liver resection was very low. Nearly all the patients had R0 resection. Only one patient with diffuse NELM had margin involvement at the histopathology report. Although high mitotic figures and increased in Ki-67 expression might provide prognostic value in patients with NELMs in the literatures, we did notice statistical significance in our current study. It probably might be due to the relatively small number of patients being investigated in this series. Rosenau et al. suggested that a low ki-67 (<5%), e-Cadherin and p53 expression had better survival performance when compared to those with high Ki-67, e-Caherin and p53 expression in patients who had undergone liver transplantations [8]. Cho et al. demonstrated that patients with high grade tumour (>50 mitotic figures at 50x high power field and/or presence of extensive necrosis) would have poorer survival performance. The median survival for patients with high grade tumour was 6 months only [23]. The pathological examination of NET should involve an expert pathologist who had enthusiasm in performing all these special staining. However, due to scarcity of this disease, the reporting of these pathological features was hardly standardized. These special pathological details were seldom presented in most of the published literatures [2, 13, 24]. Therefore the importance of these prognostic indicators should not be over stressed and hindered in the consideration of radical liver resection. Hepatectomy after all provided the best hope of cure in patients with resectable NELMs.

Univariate analysis for potential risk factors affecting survival was performed in this study. Although none of the factors demonstrated statistical significance in affecting survival, factors like tumor number and mitotic activity came close to meeting significance. We could not demonstrate the detrimental effect of these two factors because the study was limited by the relatively small sample number due to rarity of the disease. High mitotic activates and multifocal liver metastases might be poor prognostic factors in patients receiving hepatectomy for NELM.

Since not every patient was a suitable candidate for liver resection and the tumour response rate varies a lot in IAT, there had been a lot of enthusiasm in the study of nonsurgical or nonliver directed therapies. Nonetheless, there was never a large scale randomized control trial on the treatment of NET or NELM due to the scarcity of the disease entity. Most of the evidence was gathered from small scale retrospective studies. Management of NETs should involve a multidisciplinary team as different treatment strategices could be employed in different disease entities and presentations.

## 6. Conclusion

Hepatectomy is a safe and effective option for NELM. Reasonable outcome on long term overall survival and disease-free survival can be achieved with a low morbidity rate.

## Figures and Tables

**Table 1:** Demographic data of patients before hepatectomy.

Variables	(*n* = 12)
Sex (M : F)	7 : 5
Age (median (range))-liver resection	48.5 (20–71)
HBsAg positive	2 (16.7%)
Serum bilirubin level (umol/L)	8.5 (4–22)
Serum AST	30 (17–203)
Serum ALT	28 (16–65)
INR	1 (0.9–1.2)
Albumin (g/L)	41 (35–46)
PT (second)	12.1 (10.2–14.9)
Creatinine level (umol/L)	80 (58–131)
Hemoglobin level (g/dL)	12.6 (9.3–14.9)
No. of patients with comorbid disease(s)	5 (41.7%)
(i) Cardiovascular disease	9 (75%)
(ii) Pulmonary disease	1 (8.3%)
(iii) Renal impairment	0 (0%)
(iv) Diabetes mellitus	2 (16.7%)
(v) Grave disease	1 (8.3%)

**Table 2:** Operations details of liver resections for NELM.

Variables	(*n* = 12)
Blood loss (mL)	1.29 (0.2–7.0)
Hospital mortality (%)	0 (0%)
Hospital stay (days)	11 (4–56)
Operation time (minutes)	418 (195–660)
No. of patients with complications	3 (25.0%)
Pleural effusion with tapping	1 (8.3%)
Pleural effusion without tapping	1 (8.3%)
Subphrenic collection with tapping	1 (8.3%)
Clavien Dindo classification	
3A	3
3B	0
4A	0
4B	0
5	0
Tumor size (cm)	8.75 (0.9–21.0)
Tumor number	
1	6 (50.0%)
3	1 (8.3%)
4	1 (8.3%)
Multiple	4 (33.3%)
Margin size (cm)	0.5 (0.1–1.5)
Margin involved	1 (8.3%)
Presence of microvascular invasion	6 (50%)

**Table 3:** Summaries of the 12 patients with NELM.

Patient	Age	Sex	Tumour origin	Number of tumours	Type of liver resection	Mitotic figures	Ki-67 expression >5%	Survival (months)	Deceased/Alive
1	42	F	Pancreas	Multiple nodules	Extended left hepatectomy	NA	No	25.11	Deceased
2	52	F	Unknown	1	Right trisegmentectomy	Low	No	57.80	Deceased
3	45	F	Unknown	1	Right trisegmentectomy	High	No	4.04	Deceased
4	58	M	Pancreas	Multiple nodules	Wedge resection	Low	No	52.84	Deceased
5	37	M	Unknown	1	Left hepatectomy	Low	Yes	31.51	Alive
6	50	F	Pancreas	Multiple nodules	Left lateral sectionectomy and caudate lobectomy	Low	Yes	16.27	Deceased
7	45	M	Adrenal	Multiple nodules	Left hepatectomy	Low	No	14.46	Deceased
8	66	M	Pancreas	4	Right hepatectomy	NA	No	21.82	Alive
9	67	M	Unknown	1	Wedge resection	Low	Yes	51.07	Alive
10	71	M	Pancreas	3	Right hepatectomy	NA	No	13.64	Deceased
11	47	M	Colon	1	Wedge resection	Low	No	32.47	Alive
12	20	F	Pancreas	1	Extended right hepatectomy	NA	No	24.88	Alive

**Table 4:** Univariable analysis of factors affecting overall survival.

	Median survival (se)	*P*-value
Age (median)		
≤48.5 (*n* = 6)	25.11 (10.65)	0.590
>48.5 (*n* = 6)	52.84 (27.94)	
Sex		
Male (*n* = 7)	52.84 (0)	0.651
Female (*n* = 5)	25.11 (7.04)	
Blood loss (median)		
≤1.285 L (*n* = 6)	>51.07 (—)	0.590
>1.285 L (*n* = 6)	25.11 (10.65)	
Postoperative complication		
No (*n* = 8)	25.11 (17.47)	0.799
Yes (*n* = 3)	>51.07 (—)	
Magnitude of operation		
Major resection (*n* = 9)	25.11 (8.94)	0.314
Minor resection (*n* = 3)	52.84 (—)	
No. of tumors		
Solitary (*n* = 6)	57.81 (0)	0.072
Multiple (*n* = 6)	16.27 (5.22)	
Median tumor size		
≤5 cm (*n* = 5)	16.27 (1.98)	0.327
>5 cm (*n* = 7)	52.84 (21.61)	
Mitotic Figure		
Low (*n* = 7)	>51.07	0.057
High (*n* = 1)	4.042	
Microvascular invasion		
Absent (*n* = 5)	>51.07 (—)	0.367
Present (*n* = 6)	25.11 (10.65)	
Resection margin		
R0 resection (*n* = 10)	57.81 (0)	0.612
R1 resection (*n* = 1)	25.11 (—)	
